# Model-based adaptive phase I trial design of post-transplant decitabine maintenance in myelodysplastic syndrome

**DOI:** 10.1186/s13045-015-0208-3

**Published:** 2015-10-23

**Authors:** Seunghoon Han, Yoo-Jin Kim, Jongtae Lee, Sangil Jeon, Taegon Hong, Gab-jin Park, Jae-Ho Yoon, Seung-Ah Yahng, Seung-Hwan Shin, Sung-Eun Lee, Ki-Seong Eom, Hee-Je Kim, Chang-Ki Min, Seok Lee, Dong-Seok Yim

**Affiliations:** Department of Pharmacology, College of Medicine, The Catholic University of Korea, 222 Banpo-Daero, Seochogu, Seoul, Republic of Korea; PIPET (Pharmacometrics Institute for Practical Education and Training), 222 Banpo-Daero, Seochogu, Seoul, Republic of Korea; Catholic Blood and Marrow Transplantation Center, Seoul St. Mary’s Hospital, The Catholic University of Korea, 222 Banpo-Daero, Seochogu, Seoul, Republic of Korea

**Keywords:** Model-based drug development, Adaptive design, Myelodysplastic syndrome, Population pharmacokinetics-pharmacodynamics, Phase I clinical trial

## Abstract

**Background:**

This report focuses on the adaptive phase I trial design aimed to find the clinically applicable dose for decitabine maintenance treatment after allogeneic hematopoietic stem cell transplantation in patients with higher-risk myelodysplastic syndrome and secondary acute myeloid leukemia.

**Methods:**

The first cohort (three patients) was given the same initial daily dose of decitabine (5 mg/m^2^/day, five consecutive days with 4-week intervals). In all cohorts, the doses for Cycles 2 to 4 were individualized using pharmacokinetic-pharmacodynamic modeling and simulations. The goal of dose individualization was to determine the maximum dose for each patient at which the occurrence of grade 4 (CTC-AE) toxicities for both platelet and neutrophil counts could be avoided. The initial doses for the following cohorts were also estimated with the data from the previous cohorts in the same manner.

**Results:**

In all but one patient (14 out of 15), neutrophil count was the dose-limiting factor throughout the cycles. In cycles where doses were individualized, the median neutrophil nadir observed was 1100/mm^3^ (grade 2) and grade 4 toxicity occurred in 5.1 % of all cycles (while it occurred in 36.8 % where doses were not individualized). The initial doses estimated for cohorts 2 to 5 were 4, 5, 5.5, and 5 mg/m^2^/day, respectively. The median maintenance dose was 7 mg/m^2^/day.

**Conclusions:**

We determined the acceptable starting dose and individualized the maintenance dose for each patient, while minimizing the toxicity using the adaptive approach. Currently, 5 mg/m^2^/day is considered to be the most appropriate starting dose for the regimen studied.

**Trial registration:**

Clinicaltrials.gov NCT01277484

## Background

DNA methylation is the best-known epigenetic marker for cancer development [1]. In some hematologic malignancies including myelodysplastic syndrome (MDS), DNA methylation results not only in increased cell proliferation but also in silencing of genes which regulate growth and differentiation [2]. Based upon those mechanisms, the use of a DNA hypomethylating agent (HMA) for hematologic malignancies has been expanded. Accordingly, clinical researches to optimize HMA therapy [3, 4] or to explore epigenetic mechanisms for new drug development have been widely performed [5, 6].

Decitabine (Dacogen®, 5-aza-2′-deoxycytidine) is a HMA that exerts its antitumor activity by inhibiting DNA methylation at low doses and by arresting DNA synthesis at high doses [7, 8]. For several decades, decitabine has been one of the most intensely studied anticancer agents in the field of hematology due to its sophisticated development history [9–11], as well as its impressive clinical outcomes against many hematologic diseases [7, 9, 12–17]. For MDS, the approved indication for decitabine, numerous efforts have been made to optimize the dosing regimen according to patient characteristics, including the regimen evaluated in this study (five consecutive days of dosing with 4-week interval) [12, 18–22].

Recently, HMA maintenance therapy after allogeneic hematopoietic stem cell transplantation (allo-HSCT) has been suggested as a potentially attractive approach to minimize relapse and to improve graft survival [23–25]. Several studies on azacitidine (Vidaza®, 5-azacytidine) reported low toxicity, along with its potential to increase the number of hematopoietic stem cells [11, 26–29]; thus, similar approaches using decitabine were initiated [22]. In this context, we designed and performed a phase I study that aimed to find a clinically applicable dosage regimen for decitabine maintenance treatment after allo-HSCT in patients with higher-risk MDS and secondary acute myeloid leukemia (AML).

Our study design incorporated two major considerations: (1) the purpose of the maintenance therapy was to maintain disease-free status in the patient while simultaneously preserving graft function, and (2) the dosage regimen should be determined using the smallest number of patients possible. Considering these aspects, without a confident estimation of the appropriate starting dose, traditional fixed-dose escalation schemes [30] were considered inappropriate for the following reasons: (1) fatal toxicity (e.g., graft failure) might occur in some subjects, (2) the study might need too many patients to find the optimal dose [31], and (3) dose differences between cohorts might be too large or small. Thus, we introduced an adaptive dose individualization design based upon pharmacokinetic (PK)-pharmacodynamic (PD) modeling for the neutropenia and thrombocytopenia caused by decitabine.

Dose individualization of anticancer drugs using PK-PD modeling has been theoretically proposed using simulated data [32, 33]; however, our report is the first to implement dose individualization using PK-PD modeling in patients in a phase I clinical trial. We endeavored to titrate the appropriate dose for each patient, with the goal of identifying the highest possible dose that did not result in severe hematologic toxicities. We also anticipated that this approach would more quickly accomplish the study’s objectives and avoid having to test several cohorts for the dose escalation. This report focuses on the study design, the PK-PD model development for hematologic toxicities caused by decitabine, and the usefulness of our adaptive approach as it applies to subject safety.

## Results

### Patient characteristics

Five patients with secondary AML evolving from MDS and 11 with MDS (9 males, 7 females) were enrolled (Table 1). All the patients received the myeloablative condition regimen and peripheral blood stem cells from the related (*n* = 6) or unrelated (*n* = 10) donors. The engraftment achievement of platelet and neutrophil counts was confirmed for all patients by an experienced hematologist upon enrollment. Graft-versus-host disease (GVHD) prophylaxis was calcineurin inhibitors (cyclosporine for related and tacrolimus for unrelated donors) plus short-course methotrexate. Antithymocyte globulin was given to all patients. Decitabine was administered at a median of 86 days (range, 56–90 days) after transplantation. At the time of decitabine treatment, acute (≤ overall grade 2) or chronic GVHD was observed in nine and one patients, respectively. The clinical features are given in Table 2.

**Table 1:** Patient demographics

Variables	Cohort					Total
	1	2	3	4	5	
Age (year)	41.0 ± 17.1	57.0 ± 12.1	55.7 ± 5.8	55.3 ± 11.7	39.7 ± 17.9	49.2 ± 14.4
Sex (male/female)	3/1	2/1	0/3	3/0	1/2	9/7
Height (m)	1.68 ± 0.05	1.65 ± 0.10	1.58 ± 0.00	1.71 ± 0.05	1.65 ± 0.14	1.65 ± 0.08
Weight (kg)	55.4 ± 5.5	65.0 ± 13.1	52.1 ± 1.8	69.4 ± 5.0	60.3 ± 8.6	60.1 ± 9.2
Body surface area (m^2^)	1.6 ± 0.1	1.7 ± 0.2	1.5 ± 0.0	1.8 ± 0.1	1.7 ± 0.2	1.7 ± 0.2

**Table 2:** Patient characteristics and doses given in each subject, cohort, and cycle

Cohort	Subject number	Sex/age	WHO diagnosis	Donor	GVHD grade^a^		Cycle (mg/m^2^/day for 5 days)			
					Acute	Chronic	1	2	3	4
1	1	F/19	RAEB-2	MSD	0	0	5^b,c^	1.5	1.5	1.5
	2	M/36	AML	MSD	0	0	5^b^	6	5.5	6
	3	M/60	RAEB-2	MUD	1	0	5	–	–	–
	4	M/48	AML	MSD	0	0	5^b,c^	1.5	2.5	3
2	5	M/64	RAEB-2	PMUD	1	0	4^b^	4	5.5	7
	6	F/43	RAEB-2	MSD	0	0	4^b^	7	8	12
	7	M/64	AML	MSD	0	0	4^b^	6	5.5	5.5
3	8	F/51	RAEB-2	MUD	1	0	5^b^	7.5^b,c^	7.5^c^	7
	9	F/59	RAEB-1	MUD	2	0	5^b,c^	3.5	4	4.5
	10	F/36	RAEB-2	MUD	1	0	5^b^	6^b^	8.5	9
4	11	M/64	RAEB-2	MSD	2	0	5.5^b^	2^b^	–	–
	12	M/60	RAEB-2	MSD	0	Mild	5.5^b,c^	4.5^b^	7	8
	13	M/41	RAEB-2	MSD	0	0	5.5^b,c^	3	5	8
5	14	M/49	AML	MUD	2	0	5^b^	1.5	2.5	3
	15	F/50	AML	MSD	2	0	5^b,c^	4	6	9
	16	F/49	RAEB-2	MSD	2	0	5^b^	7.5	8	11^c^

*GVHD* graft-versus-host disease, *RAEB* refractory anemia with excess blast, *MSD* matched sibling donor, *PMUD* partially matched unrelated donor, *MUD* matched unrelated donor

^a^Assessed at the time of decitabine initiation

^b^Individual dose titration (IDT) by the PK-PD model was not applied

^c^The cycles where grade 4 toxicities occurred

### Patient disposition and dataset

**Fig. 1:**
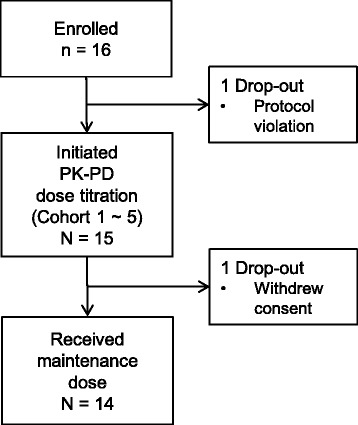
Patient disposition

Patient dispositions are detailed in Fig. 1. In cohort 1, the third patient dropped out of the study without PD sampling; thus, we substituted with an additional patient, since PK-PD results from three patients were needed to obtain the initial dose for cohort 2. Fourteen patients completed all the study-related procedures until Cycle 4, and maintenance dose was determined for each patient at the end of Cycle 4 (Table 2).

For each subject, PK sampling was performed according to the protocol, and the average number of PD observations used in individual dose titration (IDT) was 5.76/cycle for both neutrophils and platelets. Among 58 treatment cycles of 15 patients, the doses for Cycles 2 to 4 (a total of 39 cycles) were determined through PK-PD model-based adaptive dose individualization. Cycle 2 doses in four patients were clinically determined for the following reasons: no significant blood cell count decrease after cycle 1 (subjects 8 and 10) and not enough time for PK-PD modeling and IDT from sudden changes in visit schedules for Cycle 2 dosing (subjects 11 and 12). The actual dosing interval was 34.5 ± 8.7 days (mean ± SD).

### Estimated doses and safety outcomes

In all but one patient (14 out of 15), the absolute neutrophil count (ANC) was the dose-limiting factor throughout all cycles. During the cycles in which IDT was performed, the median ANC nadir observed was 1100/mm^3^ (range, 300/mm^3^ to 2680/mm^3^). The maintenance dose determined with four cycle data was higher than the initial doses in 10 out of the 15 patients. The initial doses (Cycle 1 doses) estimated by cohort dose estimation (CDE) were 4, 5, 5.5, and 5 mg/m^2^/day for cohorts 2, 3, 4, and 5, respectively. The median individual maintenance dose of decitabine was 7 mg/m^2^/day (Table 2). Maintenance doses for the patients with Cycle 1 data inadequate for PK-PD modeling could be estimated using three cycle data (Cycles 2, 3, and 4) with acceptable model fits.

A total of nine dose-limiting toxicities (DLT, platelet count for one case and absolute neutrophil count for eight cases) were observed. Among these toxicities, seven cases occurred in non-IDT cycles (six in Cycle 1 and one in Cycle 2 with clinically determined doses). In the observed toxicities, 36.8 % of the non-IDT cycles (7 out of 19 cycles) showed dose-limiting toxicities, which was an approximately seven times higher occurrence rate than that observed in the IDT cycles (5.1 %, 2 out of 39 cycles).

### Overall mixed-effect PK-PD analysis

A total of 95 PK observations and 622 PD observations (311 for ANC and 311 for platelet count, PC) were used in the overall mixed-effect PK-PD analysis. The one patient whose dose-limiting factor was PC was excluded from this analysis, whose disease entity was considered not to be similar to others, as she suffered from immune thrombocytopenia after transplantation and was managed with steroids. Among the data, 6.9 % (4 out of 58 cycles) was obtained from the cycles where IDT was not applied.

A two-compartment model was found to best describe the PK data. The between-subject variability (BSV) for *CL* (clearance from the central compartment) was the only random effect which could be estimated, except for the proportional residual error. The basic structure of the PD model was identical to that used for IDT and CDE for both PC and ANC (transit compartment model with feedback mechanism):

$$ \begin{array}{c}\frac{dA(1)}{dt}\kern0.5em =\kern0.5em {k}_{\mathrm{tr}}\cdot \kern0.5em A(1)\cdot \kern0.5em \left\{\left(1-\mathrm{SLOPE}\cdot C\right)\cdot {\left(\mathrm{BASE}/A(5)\right)}^{\mathrm{GAMMA}}-1\right\}\\ {}\frac{dA(2)}{dt}\kern0.5em =\kern0.5em {k}_{\mathrm{tr}}\cdot \kern0.5em \left(A(1)-A(2)\right)\\ {}\frac{dA(3)}{dt}\kern0.5em =\kern0.5em {k}_{\mathrm{tr}}\cdot \kern0.5em \left(A(2)-A(3)\right)\\ {}\frac{dA(4)}{dt}\kern0.5em =\kern0.5em {k}_{\mathrm{tr}}\cdot \kern0.5em \left(A(3)-A(4)\right)\\ {}\frac{dA(5)}{dt}\kern0.5em =\kern0.5em {k}_{\mathrm{tr}}\cdot \kern0.5em \left(A(4)-A(5)\right)\end{array} $$

where *A*(*N*) is the cell count in the *N*th compartment and C is the plasma decitabine concentration. A detailed description for the parameters is presented in Table 3. *BASE* is a parameter indicating the level of cell count maintained at baseline or at the period without drug effect. For platelets, an asymptotic structure describing gradual cell count increase over cycles improved the model significantly, and thus the following structure substituted the simple *BASE* parameter:

**Table 3:** Final parameter estimates and bootstrap outcomes

Parameter	Unit	Description	Population typical value		Between-subject variability	
			Estimate	Bootstrap median (95 % CI)	Estimate (as CV%)	Bootstrap median (95 % CI)
Pharmacokinetic parameters						
*CL*	L/h·m^2^	Clearance	87.8	88.3 (72.2–108)	21.4	20.5 (13.0–26.8)
*V*_c_	L/m^2^	Volume of central compartment	18.5	18.2 (14.2–23.5)	NE	NE
*V*_p_	L/m^2^	Volume of peripheral compartment	22.9	21.9 (15.7–46.1)	NE	NE
*Q*	L/h·m^2^	Intercompartmental clearance	13.1	13.3 (10.0–19.6)	NE	NE
Pharmacodynamic parameters for platelet						
*k*_tr,P_	h^−1^	Rate constant of inter-compartmental platelet movement	0.0244	0.0246 (0.0236–0.0254)	NE	NE
*SLOPE*_*P*_	–	Drug effect on platelet count	0.0656	0.0676 (0.0539–0.0930)	25.7	20.8 (1.30–56.0)
*BASE*_*P*_	/mm^3^	Baseline platelet count	49,200	53,700 (36,100–95,800)	105	78.3 (38.3–110)
*GAMMA*_*P*_	–	Shape factor for platelet count fluctuation	0.304	0.299 (0.264–0.325)	NE	NE
*IMP*		Maximum degree of platelet count recovery expected	55000	58700 (24200 – 98300)	78.3	72.9 (19.5 – 150)
*IMK*		Rate constant for asymptotic platelet count recovery	0.000530	0.000513 (0.000213–0.000691)	NE	NE
Pharmacodynamic parameters for neutrophil						
*k*_tr,N_	h^−1^	Rate constant of inter-compartmental neutrophil movement	0.0132	0.0133 (0.0120–0.0139)	NE	NE
*SLOPE*_*N*_	–	Drug effect on neutrophil count	0.263	0.237 (0.114–0.363)	57.5	58.1 (25.8–119)
*BASE*_*N*_	/mm^3^	Baseline neutrophil count	3240	3015 (2110–4140)	43.5	40.6 (27.2–57.6)
*GAMMA*_*N*_	–	Shape factor for neutrophil count fluctuation	0.193	0.181 (0.110–0.251)	39.4	36.7 (15.0–65.4)
Residual error						
*σ*_PK_^2^	–	Variance of residual error (proportional) for PK	0.441	0.436 (0.354–0.505)	–	–
*σ*_PD,P_^2^		Variance of residual error (additive) for platelet count	25,000	24,300 (18,800–29,600)	–	–
*σ*_PD,N_^2^		Variance of residual error (additive) for neutrophil count	754	748 (613–839)	–	–

Proportion of successful convergence: 78.8 % for PK model, 78.0 % for PD model

*NE* not estimated

$$ {\mathrm{BASE}}_p+\mathrm{IMP}\kern0.5em \left(1\kern0.5em {\displaystyle {-\kern0.5em e}^{-\mathrm{I}\mathrm{M}\mathrm{K}*\mathrm{TIME}}}\right) $$

where *IMP* is the empirical value of the maximum PC recovery expected, *IMK* is the rate constant for asymptotic PC recovery, and TIME is the time from the initiation of decitabine treatment.

**Fig. 2:**
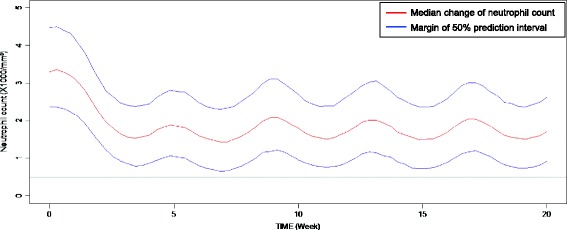
Prediction of neutrophil count change when 5 mg/m^2^ dose is given for five consecutive days with 4-week interval. (From 1000 simulations using the final PK-PD model)

No meaningful covariate was found in either the patient demographic or clinical variables. The parameter descriptions and estimates are given in Table 3. Simulated time courses of ANC changes, under the maintenance dosage of 5 mg/m^2^/day for four treatment cycles, are presented in Fig. 2.

### Clinical course and non-hematological events

During four cycles of the dose-finding phase of this study, one patient (subject 3) died of pneumonia (protocol violation) while the other two (subject 1 and subject 11) also suffered from pneumonia but fully recovered. One of the three cases developed decitabine-induced neutropenia (subject 11, withdrawn). Aggravation of existing acute or chronic GVHD was not observed, while chronic GVHD was diagnosed in two patients (one in mild and the other in moderate form). Herpes zoster was a complication in three patients.

## Discussion

We succeeded in administering the maximum dose allowed for each patient, with minimized toxicity. The dose for each cycle was determined based upon the observed cell counts in the previous cycle(s) which are the ultimate outcome of patient characteristics and drug effect. Thus, the dose can be considered as a reflection of the vulnerability of the graft, the sensitivity to decitabine, and any possible drug interactions affecting cell counts. This method meant that using a large number of cohorts, as typically required in the traditional dose escalation scheme, could be avoided. Moreover, the doses of four patients were reduced from their initial doses because of their relatively vulnerable PD characteristics. The treatment of these patients might have been discontinued if a traditional, fixed-dose design had been used. Most importantly, our study design showed the significant advantage that all dose individualization steps were accomplished with a favorable toxicity profile, judging from the proportion of cycles that exhibited grade 4 toxicities. When IDT was applied, the proportion of cycles exhibiting grade 4 toxicities dropped to approximately one-seventh the level (36.8 versus 5.1 %) compared with the non-IDT cycles. Thus, model-based dose individualization can be a useful option in early-phase clinical trial designs, in particular when the initial dose cannot be set with sufficient confidence.

The PK properties of decitabine in Korean patients obtained here are similar to those in previous studies. Liu et al. [34] and Cashen et al. [35] reported that the PK properties of decitabine could be well described with a two-compartment model. The distributional characteristics from these two studies could be indirectly compared using the maximum concentration (*C*_max_) predicted upon the completion of decitabine infusion. From previous reports, the maximum concentration of decitabine was within the range of 60–70 ng/mL, which was obtained approximately 1 h after initiation of infusion, when decitabine was administered at a rate of 5 mg/m^2^/h (3-h infusion of a 15 mg/m^2^ dose) [35, 36]. This observation is consistent with our finding that the predicted *C*_max_ after 1-h infusion of 5 mg/m^2^ was 66.0 ng/mL. In addition, the average terminal half-life was also similar (0.31 h in this study); thus, the decitabine concentration is predicted to drop below 5 % of *C*_max_ within 1.5–2 h after the completion of infusion.

The baseline cell count increase over cycles was modeled for platelet level. This was a consistent finding to the results from previous reports regarding the contribution of decitabine to cell proliferation [37–41]. For neutrophil counts, doses estimated by neutrophil count nadirs were gradually escalated over cycles until reaching the maintenance dose in ten patients while baseline cell count increase was not meaningful. Gradual deflation in the width of the prediction interval for ANC, resulting from improved precision of the model along with increased data points obtained throughout the cycles, seems to be one possible explanation. Dose escalation from this prediction interval deflation lowers the predicted median of course while maintaining the lower 25 % prediction interval above 500/mm^3^ (grade 4 toxicity).

We also found it necessary to modify the interval between cycles that was initially planned as 4 weeks in this study. Although both PC and ANC were recovered to the baseline after decitabine dosing, our PK-PD model predicted that the time to nadir was 3.5 weeks and that the time to recovery from the influence of the last dose (ANC >1000/mm^3^) was approximately 5 weeks for the ANC. This prediction was consistent with the actual dosing interval practiced in this study (34.5 days on average). This finding implies that the 4-week interval may not be long enough to initiate the next cycle. Moreover, as illustrated in Fig. 2, the lowest value of ANC appears to be achieved in the second cycle (6–7 weeks after treatment initiation). Thus, the initial nadir of ANC within the first 4-week cycle should not be mistaken for the lowest ANC value throughout the cycles. This could also have been a reason for failure in dose determination if traditional fixed-dose escalation based on the first cycle nadir was recruited. To optimize the dosing regimen that may overcome this difficult property of decitabine, an initial loading dose may be considered before giving maintenance doses.

## Conclusions

We exemplified the adaptive dose titration approach, based upon a quantitative exposure-toxicity model, in this study. This approach seemed most useful, since this method enabled rapid and precise dose individualization. The most appropriate initial dose was determined to be 5 mg/m^2^/day for five consecutive days. Throughout the course of data analysis, issues such as extending between-cycle intervals and the use of loading doses were also raised. Cohort 6 is ongoing for exploration of the adequacy of the recommended starting dose, and additional report will be provided after completion of 12 cycles of treatment of all participants.

## Methods

### Ethics, consent, and permission

This study was designed and conducted in accordance with the principles of the Declaration of Helsinki and the good clinical practice guidelines of Korea. The independent institutional review board of Seoul St. Mary’s Hospital approved this study protocol before the initiation of any study-related procedure, and written informed consent was obtained from every subject. The registration number of this trial at “ClinicalTrials.gov” is NCT01277484.

### Patient eligibility

Patients starting decitabine treatment on days 42–90 after allo-HSCT and meeting the following criteria were considered eligible: adult aged ≤65; recipient of allo-HSCT for higher-risk (intermediate 2 or high risk) MDS, as assessed by the International Prognostic Scoring System [42], and/or AML evolving from MDS; disease remission with appropriate recoveries of PC >30,000/mm^3^ and ANC >1000/mm^3^, both of which were maintained for more than 7 days without any transfusions or growth factors; absence of grade III/IV acute GVHD; Eastern Cooperative Oncology Group (ECOG) performance status of 0 to 2; and no evidence of renal or hepatic impairment.

### Study design

Patients were assigned to cohorts according to their order of enrollment. A cohort consisted of three patients to whom the same initial daily dose of decitabine (according to body surface area) was given. The initial dose for cohort 1 was 5 mg/m^2^/day. The designated dose was infused intravenously over 60 min daily for five consecutive days in each cycle, and the cycle was repeated every 4 weeks up to Cycle 12. However, dosing was suspended if blood cell counts insufficiently recovered (PC <30,000/mm^3^, ANC <1000/mm^3^).

For cycles 2 to 4, the dose for each cycle was estimated using IDT according to PK-PD modeling and simulations based on blood cell count data accumulated until the time of dose estimation (just before administration). The maximum dose at which the occurrence of grade 4 hematologic toxicity (dose-limiting toxicity, PC <25,000/mm^3^ or ANC <500/mm^3^) could be avoided at the lower limit of the 50 % prediction interval (25th percentile), according to 500 simulations, was determined to be the dose for the next cycle. If the data from the previous cycle were not adequate for PK-PD modeling (e.g., no significant blood cell count decreases), the dose was determined based upon the hematologist’s clinical decision [43]. Only the upper limit of the dose increment was pre-determined that the next cycle dose cannot exceed 150 % of the previous dose. The dose determined at Cycle 4 for each individual was maintained thereafter.

The fixed initial dose for each cohort was also estimated using PK-PD modeling and simulations and was based on the observations from the previous cohorts (CDE). For cohort 2, all of the data obtained before the initiation of treatment for the first patient in cohort 2 were used for the initial dose estimation; however, only Cycle 1 data from the previous cohorts were used for cohort 3, 4, and 5. A new cohort was not initiated before completion of the first cycle in the last patient of the previous cohort.

**Fig. 3:**
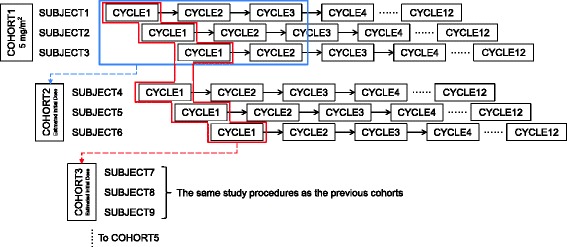
Overall schema of the study design. Individual dose titration was performed for the next cycle based on the observations from the previous cycle (*solid straight arrows*). Cohort dose estimation was performed to determine initial doses (*broken line arrows*): (i) for cohort 2, using all data obtained from cohort 1 until the initiation of cohort 2; (ii) for cohorts 3–5, using only Cycle 1 data of previous subjects. The dose of Cycle 4 was maintained until the completion of decitabine treatment (Cycle 12) (*dotted lines*)

A schematic diagram of the overall study design is presented in Fig. 3.

### PK and PD samplings

To determine plasma concentration measurements, seven whole-blood samples (10 mL each) were collected using EDTA tubes before dosing and then at 20, 40, 60, 90, 120, and 180 min after initiation of the first dose infusion of Cycle 1. The samples were immediately cooled in an ice bath and then centrifuged (3000 rpm, 4 °C, for 10 min) within 1 h from the last sampling time. After centrifugation, 4 mL of plasma from each sample was aliquoted into four microtubes (1 mL each), and 10 μL of 10 mg/mL tetrahydrouridine (THU) solution was added to each microtube. Microtubes were stored at −70 °C until plasma concentration assays.

As PD (toxicity) markers, PCs and ANCs were monitored at scheduled follow-up visits (weekly until Cycle 4 and biweekly thereafter). The procedures for obtaining PCs and ANCs followed the routine clinical practices for automated complete blood cell counts at Seoul St. Mary’s Hospital.

### Plasma concentration measurements

Plasma samples were analyzed using liquid chromatography coupled with tandem mass spectrometry (API 4000, ABSciex, Canada) based upon a previously reported method [34]. The lower limit of quantification (LLOQ) was 0.5 ng/mL. The coefficients of correlation (*r*^2^) were greater than 0.9975 in the range of 0.5–100 ng/mL decitabine, as determined by weighted linear regression (1/concentration). The precision (% coefficient of variation) and mean intra- and inter-day accuracies were below 11.57 % and 95.55–102 %, respectively.

### PK-PD modeling and simulation

A mixed-effect analysis was performed using NONMEM (ver. 7.2, Icon Development Solution, Ellicott City, MD, USA). During the early phase of this study (e.g., IDT for cohort 1 and CDE for cohort 2), during which sufficient PD data to build a robust model were unavailable, we adopted the PD model proposed by Wallin et al. (2009) [33]. This model was used in conjunction with the one-compartment, first-order elimination PK model to build the initial PK-PD model. Therefore, only the values of the PK-PD parameters for each individual were estimated at this step. Then, as data accumulated, we performed additional modeling to find a better PK-PD model structure that optimally fits the data. Multi-compartment PK models, in addition to PD structures such as baseline cell count increase, were tested in the modeling process.

Random effects were also taken into consideration. The structure to describe the residual error, which refers to the deviation of each observation from the value predicted by the PK-PD model, was initially applied to both IDT and CDE procedures as follows:

$$ {\mathrm{DV}}_{ij}={\mathrm{IPRED}}_{ij}\cdot \left(1+{\varepsilon}_{\mathrm{prop},ij}\right)+{\varepsilon}_{\mathrm{add},ij} $$

where DV_*ij*_ is the *j*th measured concentration or blood cell count in the *i*th individual, IPRED_*ij*_ is the model-predicted value for the corresponding observation (DV_*ij*_), and *ε*_prop,*ij*_ and *ε*_add,*ij*_ are the residual variabilities with means of 0 and variances of *σ*_prop_^2^ and *σ*_add_^2^, respectively. For the CDE step, BSV (*η*_*i*_) of each PK and PD parameter was tested as follows:

$$ {P}_{ij}={TVP}_j\cdot \exp \left({\eta}_i\right) $$

where *P*_*ij*_ is the *j*th model parameter in the *i*th individual and *TVP*_*j*_ is the typical value of the *j*th model parameter. The BSV for each parameter was assumed to follow a normal distribution, with a mean of 0 and differing values of variance (described using the symbol *ω*_*i*_^2^).

The first-order conditional estimation with interaction option (FOCE-I) method was used whenever applicable. Model adequacies were assessed based upon goodness-of-fit plots, likelihood ratio tests (LRT), and model stability measures (e.g., successful convergence, matrix singularity, and significant digits). Cutoff criteria incorporated a *p* value of 0.05 (e.g., 3.84 for one parameter addition, 5.99 for two) for LRT to determine statistically significant improvements in the model.

Covariate analysis was performed for potential covariates, including demographic variables (sex, age, baseline body weight, and surface area) and clinical variables (mainly results from laboratory tests). After covariate screening via visual correlation check-ups and generalized additive modeling (GAM) procedures, the variables selected from the screening were tested as fixed effects for a certain PK-PD parameter, using LRT and decreases in BSV for the corresponding parameter.
